# UVA Photoprotective Activity of Brown Macroalgae *Sargassum cristafolium*

**DOI:** 10.3390/biomedicines7040077

**Published:** 2019-09-27

**Authors:** Eka Sunarwidhi Prasedya, Sundari Maulinda Syafitri, Brigitta A. F. D. Geraldine, Candra Dwipayana Hamdin, Andri Frediansyah, Masao Miyake, Daisuke Kobayashi, Akihiro Hazama, Haji Sunarpi

**Affiliations:** 1 Bioscience and Biotechnology Research Centre, Department of Biology, Faculty of Mathematics and Natural Sciences, University of Mataram, Mataram 83115, Indonesia; ekasprasedya@unram.ac.id (E.S.P.); 2771997@gmail.com (S.M.S.); brigittageraldine28@gmail.com (B.A.F.D.G.); 2 Department of Pharmacy, Medical Faculty, University of Mataram, Mataram 83115, Indonesia; candradwipayana@unram.ac.id; 3 Pharmaceutical Institute, University of Tuebingen, 72074 Tuebingen, Germany; microbiologii@gmail.com; 4 Research Unit for Natural Product (BPTBA), Indonesian Institute of Sciences (LIPI), Wonosari 55861, Indonesia; 5 Department of Cellular and Integrative Physiology, Fukushima Medical University, Fukushima 960-1247, Japan; mm@fmu.ac.jp (M.M.); coba@fmu.ac.jp (D.K.); hazama@fmu.ac.jp (A.H.)

**Keywords:** macroalgae, seaweed, sunscreen, photoprotective, ultraviolet

## Abstract

Sunscreens today contain several synthetic UV (Ultraviolet) filter molecules to protect the skin epidermis from UV radiation damage. However, these molecules may create several negative effects on human skin. Due to this condition, there is an increase in the development of natural products to replace uses of these synthetic chemicals. Brown macroalgae *Sargassum* has been recently studied for its photoprotective activities. The purpose of this study is to investigate photoprotective activity of one of most abundant Sargassum species in Lombok coast; *Sargassum cristaefolium*. Spectrophotometry analysis with UV-VIS revealed the UV spectra absorbing capability of *Sargassum cristaefolium* (SC) in the UVA spectrum range (314–400 nm). Furthermore, spectrometry analyses with LC-MS revealed the existence of UV absorbing compound MAA-palythene. In correlation, SC ethanol extracts also demonstrate that it could protect DNA from UVA irradiation as analyzed in vitro in HeLa cell model. The effects of SC on UVA exposed-dorsal mice skin have also shown interesting results, as mice pretreated with SC before UVA exposure showed protective activity on the epidermal integrity similar as positive control. Whereas, UV exposed mice without SC or commercial products resulted in increased epidermal thickness, which is the common parameter of skin photoaging. In addition, pretreated mice with SC also show protective effects in the formation of collagen connective tissues. Overall, current results show promising photoprotective activity of SC against UV radiation. More advanced investigations of SC as a potential photoprotective agent would be reasonable for development of macroalgae-based natural skin protection products.

## 1. Introduction

Repeated ultraviolet (UV) exposure to human skin can cause severe skin damage, including photo-aging, inflammation, and photo-carcinogenesis [1,2]. UV light radiation is classified into three categories depending on wavelength ranges; UVA (320–400 nm), UVB (290–320 nm), and UVC (200–290 nm). Both UVA and UVB can injure the human skin, resulting in sunburns, actinic keratosis, photo-aging, and severe harms such as skin cancers [3,4].

The use of sunscreen as photoprotecting agents against UV radiation is becoming very popular. However, most sunscreens are composed of inorganic filters, such as Zinc Oxide (ZnO) and Titanium dioxide (TiO) which could cause negative effects in the human skin [5]. Hence, several studies suggest that skin protectors derived from natural products are potentially more suitable for the human skin [6]. Hence, the concept of complementary or alternative medicine has become more widely accepted, and there is a corresponding interest in natural remedies.

Marine life is probably the most affected environment by UV radiation. Marine organisms such as algae, corals, and crustaceans must find a way to survive such extreme environmental conditions. Hence, marine organisms are forced to develop a metabolic response as a self-defense mechanism; for example, by producing secondary metabolites that allow them to protect themselves against external factors [7]. Macroalgae are one of the most considered organisms to have huge potential as a natural source of important bioactive compounds, which have several bioactivities, such as antitumor, anti-inflammatory, antioxidant, antimicrobial, and UV radiation protective activities [8,9]. Brown macroalgae *Sargassum* has frequently been reported to exhibit anti-UV absorbing compounds [10,11]. Because of its natural environment, which is mostly exposed to UV light, macroalgae *Sargassum* produces secondary metabolites for its survival [12]. Indonesia is endowed with abundance of macroalgae such as Sargassum species; particularly *Sargassum cristaefolium*, which are commonly found in most Indonesian coastal areas. The present study therefore investigates the UV photoprotective potential of Indonesian macroalgae *Sargassum cristaefolium* ethanol extract against UV radiation. Results from this research would provide basic information regarding potentials of Lombok coast, Indonesia macroalgae for development of natural UV skin protection products.

## 2. Materials and Methods

### 2.1. Sample Collection and Extraction

Macroalgae *S. cristaefolium* were sampled from Batu Layar Coast, West Lombok (8°29′54.251″ S and 116°4′36.664″ E). The macroalgae was identified with reference to algae electronic database [13]. The macroalgae samples were rinsed with freshwater to remove sand debris. Samples were then air dried for ±5 days. After constant dry weight of the samples was obtained, samples were then ground into powder. Macroalgae powder samples were mixed with absolute ethanol solvent with 5× volume of the sample weight (*w*/*v*). The seaweed samples were washed with freshwater to remove adhering debris. The collected samples were then dried and powdered. Powder samples were mixed with absolute ethanol solvent with 5× volume of sample weight (*w*/*v*). Mixed solutions were incubated at room temperature for 48 maceration process. Every 24 h, mixed solutions were filtered with Whatman grade 1 filter papers. Furthermore, macroalgae extract was subjected to rotary evaporator to evaporate the remaining ethanol solutions. Obtained filtrates were then used as macroalgae ethanol extracts for further experiments.

### 2.2. UVA–UVB Absorbing Activity

SC extract was diluted to 0.1% concentration for evaluation of its UV light absorbing capability. Extract was added in to a quartz cuvette, and its absorption spectra (wavelengths 200–800 nm) were acquired using a Nanodrop 2000c UV-Visible spectrophotometer (Thermofisher Scientific, DE, USA) against a blank containing ethanol.

### 2.3. Liquid Chromatography-Mass Spectrometry (LC-MS)

Screening of bioactive compounds in macroalgae ethanol extracts were performed by LC-MS analyses on a LCMS-Q-TQF system (Dionex, Ultimate 3000, Bruker, mi-crOTOF Q) (Bruker Daltonik GmbH, Bremen, Germany). Liquid chromatography separation was achieved, followed by 20 μL injection of extract on a C18 column (250 × 4.6 mm, 5 μm) at a flow rate of 1.4 mL·min^−1^ with a total run of 45 min. The mobile phase consisted of solvent A, methanol/water/acetonitrile 51/13/36 with 0.3 M ammonium acetate, and solvent B was methanol. The source conditions were the following: nebulizer 40 pst, dry gas 91 min^−4^, and temperature 200 °C with a scan range of 50–1000 *m*/*z*.

### 2.4. Cell Culture

HeLa cell line (DMEM, Wako, Japan) was used as in vitro cell model to evaluate macroalgae cytoprotective activity. Cells were cultured in Dulbecco’s Modified Eagle’s Medium (DMEM, Wako, Japan) supplemented with 10% Fetal Bovine Serum (FBS) in CO_2_ humidified incubator (37 °C; 5% CO_2_). Cells were grown until 80–90% confluency before used in cytoprotective assay. Cell images were obtained from phase-contrast microscopy by BZ-9000 microscope (Keyence, Osaka, Japan).

### 2.5. Cytoprotective Activity of SC Ethanol Extract against UV-A Irradiation

Human cell model HeLa cells were plated in 35 mm dishes (1 × 10^5^ cells/dish). The DMEM media was removed 24 h after seeding (except for the negative control group) and the cells were exposed SC ethanol extract (0.01%) and Ascorbic acid (0.01%) in 10% FBS supplemented DMEM medium as positive control. After this treatment, the cells were stained with DAPI (1 μg/mL). UV-A radiation was applied to the cells simultaneously with photos taken every 30 s under fluorescence microscope. Number of dead cells (excited by DAPI) were counted and analyzed with ImageJ.

### 2.6. Animals

For this experiment, a total of 30 female BALBL/c mice, 6–8-week-old and weighing 25–30 g were used. The mice had free access to food and water in a temperature (24–25 °C) and humidity (50%) controlled room. Animals were also subjected to a 12 h light/dark cycle. All experimental procedures were in compliance with the Health guidelines for Care and Use of Laboratory Animals and were approved on 13 June 2019 by the Bioethics Committee of Medical Faculty University of Mataram (Approval code: 117/UN.18.F7/E7/ETIK/2019).

### 2.7. In Vivo UV-A Irradiation

Mice were randomly divided into 4 groups (*n* = 6 per group) that represented treatments and controls. These were: normal healthy controls with no UV treatment (Control − UV), normal healthy controls with UV treatment (Control + UV), UV irradiation with 0.1% SC treatment (SC + UV), and UV irradiation with commercial product (CP + UV). Before experimentation, mice were anesthetized by intraperitoneal injection of 1% sodium pentobarbital, and hair was removed from the back skin of mice using hair removal wax.

Parallel UV lamps Reptile UV150 PT2188 13W (Exo Terra, Winchester, MA, USA) were used for UV-A irradiation treatment. Distance of UV lamp to mice was 20 cm to obtain UVA spectral radiance of 300 μW/cm^2^. Animals were irradiated for 7 days, 2 h/day, with a daily dose of 2.16 J/cm^2^.

### 2.8. Histological Measurements

Skin specimens were soaked in 10% formalin and embedded in paraffin. Approximately five micrometer thin sections of the paraffin-embedded skin were cut and subjected to hematoxylin eosin (HE) and Van Gieson (VG) histochemical methods. Morphological analysis of the histological sections was performed by bright field microscopy (Zeiss, Axio Observer D1, Oberkochen, Germany).

### 2.9. Statistical Analysis

Data were expressed as mean ± standard error of the mean (SEM) or ± standard deviation (SD). The data obtained were subjected to one-way analysis of variance (ANOVA), followed by Tukey’s test. Data analyses were conducted with GraphPad Prism (ver 5.0, San Diego, CA, USA) and Kaleidagraph (ver 4.5.2, Reading, PA, USA). Differences were considered significant if *p* < 0.05.

## 3. Results

### 3.1. Collection of Macroalgae Sargassum cristaefolium

**Figure 1:**
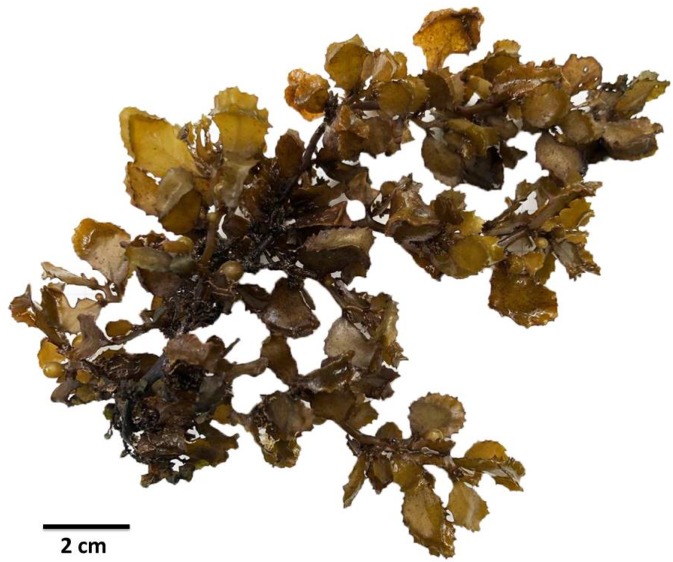
Brown macroalgae *Sargassum cristaefolium*.

Macroalgae *S. cristaefolium* were collected from North West Lombok coastal area (8°24′11.7396″ S, 116°4′1.9056″ E), West Nusa Tenggara Province, Indonesia in the early 2019 (Figure 1). Macroalgae specimens were identified according to electronic algae database [13].

### 3.2. UV Spectra Absorbance of SC Extract

**Figure 2:**
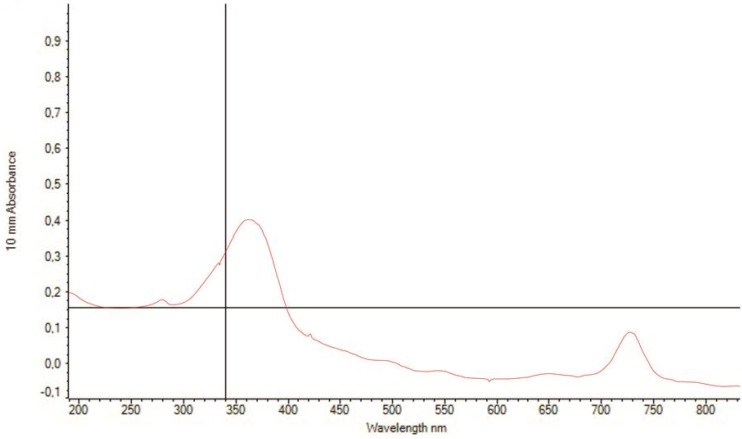
Ultraviolet Absorption spectrum of the ethanol extract of *Sargassum crassifolium* (0.1%).

The UV-Vis absorbance spectra of *Sargassum cristaefolium* (SC) ethanol extract were evaluated within the range of 200–700 nm. Figure 2 shows the UV-Vis absorbance spectra of SC shown UV absorbing capability in UV-A spectra region (340–400 nm). This result implies that brown macroalgae *S. cristaefolium* potentially exhibits UV-A light absorbing activity.

### 3.3. LC-MS, Detection of Potential UV-Absorbing Compounds

**Figure 3:**
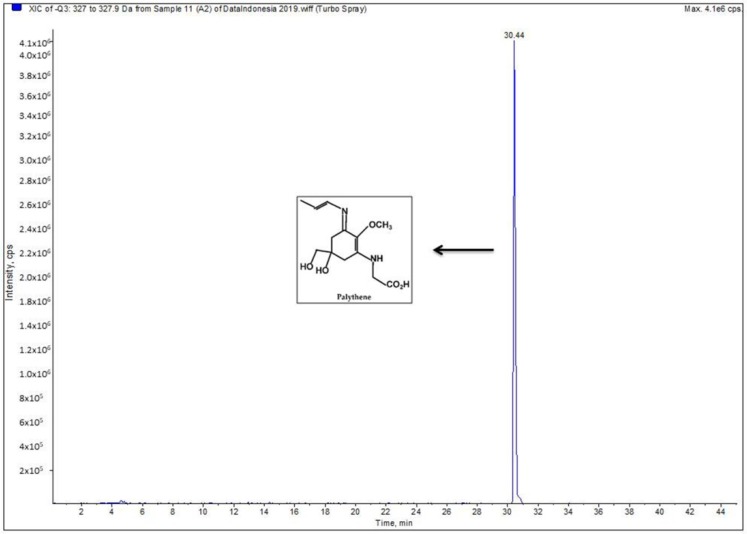
LC/MS analysis of *Sargassum cristaefolium* ethanol extract (0.1%).

The SC ethanol extract was subjected to LC-MS analysis. The mass spectrum revealed one dominant charged ion peak (Figure 3). The detected peak showed a large absorption in the region of Microsporine-like Amino Acids (MAAs), and the ion of (M-H) of 327.2 *m*/*z* corresponded to the monoisotopic molecular mass of the proton adduct of palythene [14].

### 3.4. DAPI Fluorescence Staining

**Figure 4:**
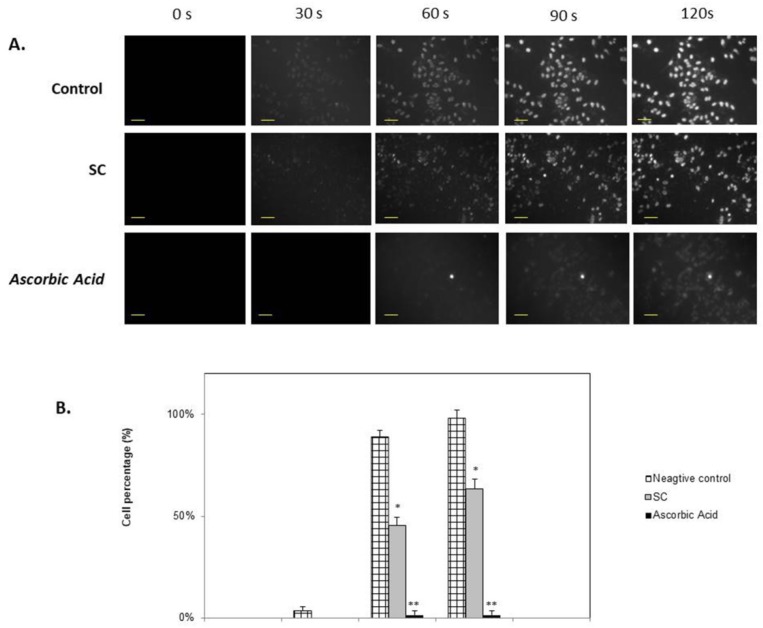
Cytoprotective analysis of macroalgae extracts (0.01%) against UV-A irradiation in HeLa cells for 2.5 min irradiation. (**A**) Nucleic staining with DAPI was conducted to determine cellular damage. Scale bar: 200 µm (**B**) Dead cell percentage of HeLa cells irradiated with UVA. * is considered significantly different to control (*p* < 0.05); ** is considered highly significantly different compared to control (*p* < 0.01).

Nucleic staining with DAPI was conducted to estimate cytoprotective activity of *Sargassum crassifolium* against UV radiation treatment. Control cells were incubated with no SC extracts, and these cells showed more intense excitation of UV light compared to SC and Ascorbic acid treated cells (Figure 4). Cells treated with SC were seen to show lower exitation of DAPI fluorescence staining when exposed to UV light. This difference was signifcantly observable at 90 s exposure compared to control, indicating photoprotective activity of SC against UVA light exposure.

### 3.5. SC Protects Skin from UVA-Induced Damage

**Figure 5:**
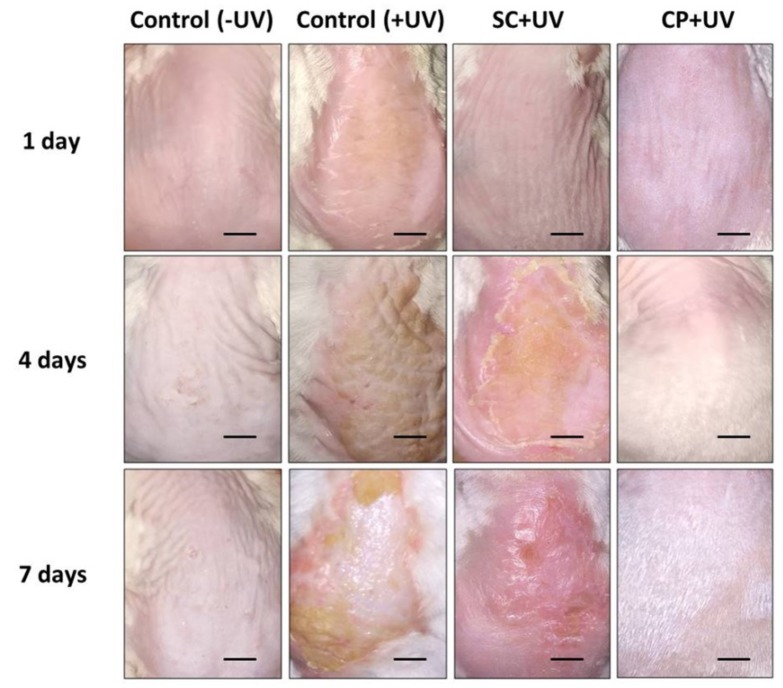
Effect of *Sargassum cristaefolium* extract (SC) on UV-irradiation skin lesion in mice for seven days. Scale bar: 1 cm.

We have verified the photoprotective activity of SC ethanol extract against UV damage in vivo. The results show that the skin in the SC group formed an oily layer that was absorbed by the skin approximately after 1 day treatment (Figure 5). There were redness and inflammation effects observed on the fourth day of treatment. However, the UVA induced damage was seen to heal on the seventh day of the treatment. Compared to the control group (Control + UV) exposed by UV, the skin was rougher and also had scaling, also there was mild congestive inflammation. Commercial product with 30 SPF (CP + UV) group shows impressing photoprotective activity, as pathological changes of the skin were almost absent and similar to control group (Control − UV) not treated with UV. Furthermore, we also observed the increase growth of hair in the dorsal area of the CP + UV group.

### 3.6. Pathological Changes of UVA-Irradiated Mice Skin Treated with SC

**Figure 6:**
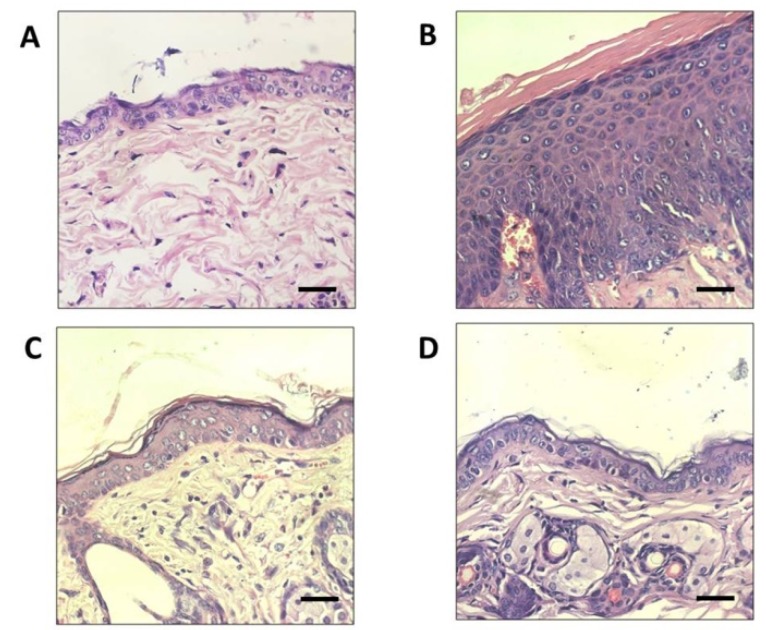
Histological analyses of mice skin irradiated by UV for two hours every day for seven days. (**A**) Control not irradiated with UV (Control − UV) (**B**) Control irradiated with UV (Control + UV) (**C**) Irradiated skin treated with 0.1% SC (SC + UV) (**D**) Irradiated skin treated with commercial product 30 SPF (CP + UV). Skin sections were stained with Hematoxylin and Eosin (H&E). Scale bars: 20 μm.

**Figure 7:**
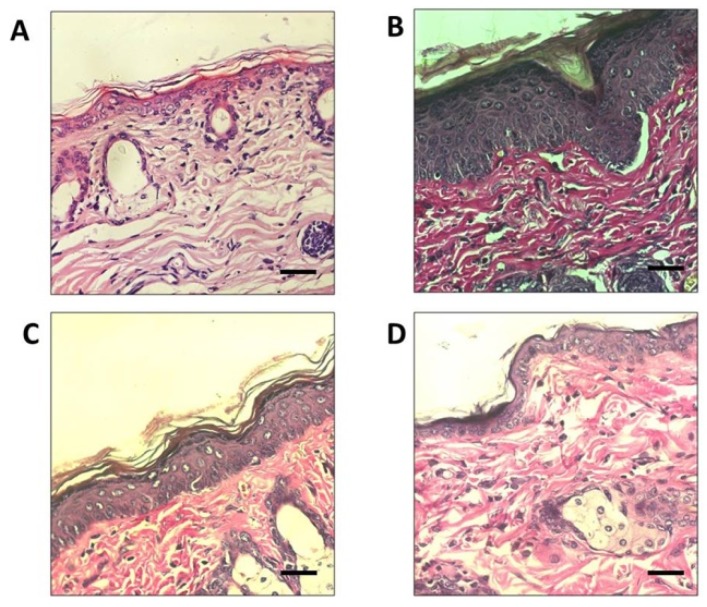
Histological analyses of mice skin irradiated by UV for two hours every day for seven days. (**A**) Control not irradiated with UV (Control − UV) (**B**) Control irradiated with UV (Control + UV) (**C**) Irradiated skin treated with 0.1% SC (SC + UV) (**D**) Irradiated skin treated with commercial product 30 SPF (CP + UV). Skin sections were stained with Van Gieson. Scale bars: 20 μm.

Pathological changes, such as epidermal thickness, could be used to evaluate effects on skin inflammation and photo-aging. In this study, the protective effects of SC were evaluated by analysis of epidermal thickness of UVA-irradiated mice (SC + UV) skin and compared with unexposed mice (Control − UV) skin. Histological analysis of dorsal skin with Hematoxylin and Eosin (H&E) staining revealed that the epidermal thickness was increased in the UVA-irradiated dorsal skin of the control group (Control + UV) exposed to UVA without SC or CP treatments (Figure 6). UV exposed groups: SC + UV and CP + UV groups showed considerable photoprotective activity, as both could maintain the integrity of the skin epidermal layer. The tissue sections were also subjected to Van Gieson staining to visualize changes in collagen fiber formation in the dermal areas of the UV-A exposed dorsal skin (Figure 7). Notably, the collagen fibers in the Control + UV group were disorganized and mostly were degraded non-functional fibers. Compared to SC + UV and CP + UV treated group, the collagen formation were neatly and densely arranged, similar to Control − UV group.

## 4. Discussion

UVA radiation effects are often overlooked, yet it produces the same hazardous effects on the skin, including sunburn, photo-aging, and skin cancer as UVB [15,16,17]. Skin protection products such as sunscreens have become an alternative to reduce damaging effects of UV radiation. However, most skin protection products rely on photoprotective activity of synthetic compounds, which potentially induces negative side effects [18]. Hence, there is an increasing interest in developing skin protection products derived from natural resources. Many studies have reported the potential of marine algae as a principle source of various bioactive substances with significant medicinal and nutritional value, such as brown macroalgae *Sargassum*, which are commonly exposed to extensive amount of UV radiation throughout the year. In this study, we evaluated the photoprotective potential of the abundant *Sargassum* species in Lombok coastal area; *Sargassum cristaefolium*.

Its capability to survive in such extreme conditions is reportedly due to the existence of broad and diverse group secondary metabolites [19]. Furthermore, Macroalgae are well reported to exhibit several UV-absorbing compounds [20], such as phlorotannins, a marine algal polyphenol which has demonstrated anti-UV activity [21]. Another common UV-absorbing compound found in marine macroalgae are the Microsporin-like Amino Acids (MAAs) [22]. Based on UV-VIS and LC-MS spectrometry analyses, brown macroalgae *S. cristaefolium* ethanol extract potentially contains MAAs Palythene (Figure 3). MAAs such as Porphyra-334, palythine, palythene, and shinorine exist in various seaweeds [23]. The presence of this compound could be the answer to the photoprotective activity of *S. cristaefolium*. Overall, macroalgae *S. polycystum* exhibits a similar qualitative phytochemical profile to *S. crassifolium*. Numerous studies have shown that MAAs prevent UVR-induced damage at the cytoplasmic level. Cells with high concentrations of MAA are likely to be more resistant to UVR than those with low concentrations [24].

Low concentration of fluorescence stain DAPI is semi-permeable to the cells. Hence, it would mostly bind to the nuclear DNA of dead cells [25]. Treatment of SC in cells resulted in significantly lower DAPI excited cells compared to untreated cells (control + UV), as shown in Figure 4. This indicates potential UVA photoprotective activity of SC ethanol extracts. Macroalgae *Sargassum muticum* have demonstrated cellular DNA protective activity against UV-A radiation [11]. Other considerable studies have also stated the photoprotective activity of brown macroaglae *Sargassum* species [26,27,28]. However, to our knowledge, this is the first study to state the photoprotective activity of *Sargassum cristaefolium*.

Continuous exposure of UV light could induce skin photo-aging, which is characterized by pathological changes in the components of the dermal connective tissue extracellular matrix, such as wrinkles and reduced amounts of collagen content [29]. Previous studies have revealed that photodamaged skin is also associated with increased epidermal thickness and dryness [30]. Within the dermal ECM, aging is associated with a thickening of collagen connective tissues and disorganization of total collagen content, mainly due to decreased collagen synthesis and increased fibril fragmentation [31]. In the present study, collagen fiber formations were seen to be restored in UVA-treated SC-pretreated skin, compared with the damaged skin of the UVA-treated CP-pretreated skin. Furthermore, on the seventh day, the SC pretreated mice group showed healing activity in UVA irradiates skin area. This result implies that SC photoprotective activity could protect collagen connective tissue from UV radiation damage.

## 5. Conclusions

In conclusion, the results of the current study suggest the cytoprotective effects of macroalgae *Sargassum cristaefolium* (SC) in the region of UV-A spectrum. Cytoprotective mechanisms of action of SC ethanol extract may correlate to presence of MAA-palythene, which probably contributes in inhibition of cellular DNA damage by UVA radiation. Furthermore, SC pretreated mice skin resulted in less pathological changes compared to untreated mice after UVA radiation. SC pretreated mice show less epidermal thickening and protects collagen connective tissue formations. Nevertheless, it could be concluded that SC may be a suitable candidate as a source of photoprotective agents with cosmeceutical and pharmaceutical value.
